# Using the Affiliate Stigma Scale with caregivers of people with dementia: psychometric evaluation

**DOI:** 10.1186/s13195-016-0213-y

**Published:** 2016-10-26

**Authors:** Chih-Cheng Chang, Jian-An Su, Chung-Ying Lin

**Affiliations:** 1 Department of Psychiatry, Chi Mei Medical Center, Tainan, Taiwan; 2 Health Service and Population Research Department, Institute of Psychiatry, Psychology and Neuroscience, King’s College London, London, UK; 3 Department of Senior Citizen Service Management, College of Recreation and Health Management, Chia Nan University of Pharmacy and Science, Tainan, Taiwan; 4 Department of Psychiatry, Chang Gung Memorial Hospital at Chiayi, Chiayi, Taiwan; 5 Graduate Institute of Clinical Medical Sciences, Chang Gung University, Taoyuan, Taiwan; 6 Department of Nursing, Chang Gung Institute of Technology, Taoyuan, Taiwan; 7 Department of Rehabilitation Sciences, Faculty of Health and Social Sciences, The Hong Kong Polytechnic University, Hung Hom, Hong Kong

**Keywords:** Affiliate stigma, Caregiver, Confirmatory factor analysis, Dementia, Rasch

## Abstract

**Background:**

In this study, we examined the psychometric properties of the Affiliate Stigma Scale to measure affiliate stigma for caregivers of family members with dementia, a topic scantily covered in the literature.

**Methods:**

Two hundred seventy-one caregivers were recruited. Each completed the Affiliate Stigma Scale, Caregiver Burden Inventory, Taiwanese Depression Questionnaire, Beck Anxiety Inventory, and 28-item World Health Organization Quality of Life questionnaire. The data were evaluated for internal consistency and concurrent validity, and they were analyzed using Rasch statistics and confirmatory factor analysis (CFA).

**Results:**

CFA and Rasch analysis suggested that the Affiliate Stigma Scale contains three underlying unidimensional concepts (cognition, affect, and behavior). The three concepts had satisfactory internal consistency (α = 0.822–0.855) and concurrent validity (*r* = 0.290–0.628 with caregiver burden, 0.391–0.612 with depression, 0.367–0.467 with anxiety, and −0.590 to −0.365 with quality of life).

**Conclusions:**

The Affiliate Stigma Scale is a promising instrument with sound psychometric properties for measuring affiliate stigma. Healthcare providers might want to use it to understand the caregivers’ perspectives and to design appropriate interventions to decrease their affiliate stigma.

## Background

Since Goffman [1] defined stigma as an attribute deeply discrediting within a particular social interaction, studies on stigma have dramatically grown [2, 3]. Additionally, different types of stigma have been defined. Structural stigma, or the imbalances and injustices in social structures, often results in poor-quality healthcare and inadequate professional behavior for stigmatized individuals [4]. Public stigma, the negative reactions from any general population toward a stigmatized group, forces stigmatized individuals to perceive and experience stereotypes of themselves [5]. Self-stigma, the internalization of the public stigma into the stigmatized individuals themselves, is attributable to the psychological effect of reflecting on having a social stigma [2] and increases social withdrawal for the stigmatized individuals [6].

In addition to the aforementioned stigma types, Corrigan et al. [7] stated that prejudice and discrimination are extended to people without the manifest marks of stigmatized characteristics because of their relationship to a person with the stigmatized mark. This kind of stigma among family members, also known as *courtesy stigma*, is called *affiliate stigma* when it is internalized [8]. On the basis of the definition, we believe that stigma among family caregivers of persons with dementia is just due to the association with their family member who has dementia. The stigma of the caregivers is not just due to the physical burden of caregiving, although the caregivers may encounter more care burden if stigma works [9]. Because of the negative consequences of affiliate stigma (e.g., unhappiness, helplessness, and negative emotions) [8, 10], many studies of their mechanisms have been done, but they are almost exclusively in connection with patients who have HIV [11] or mental disorders—especially schizophrenia [6]. Specifically, most studies [12–14] that examine the effects of courtesy stigma and affiliate stigma on caregivers of people with dementia use qualitative instead of quantitative analysis. One major reason for this is the dearth of appropriate instruments for assessing the affiliate stigma of the caregivers.

Our review of the literature revealed that four studies [3, 8, 10, 15] have developed instruments to assess affiliate stigma, but only Werner et al. [3] included patients with dementia when developing their instrument. Although Werner et al. [3] designed and validated the Family Stigma in Alzheimer’s Disease Scale (FS-ADS) using comprehensive reviews and adequate statistical methods, there are still some gaps with the FS-ADS in practical clinical use. First, the FS-ADS was designed specifically for patients with Alzheimer’s disease (AD), and its psychometric results were derived from a population with AD; thus, it might not be applicable for people with other types of dementia. Second, the participants in the Werner et al. study were all children of the people for whom they were caregivers; thus, the findings might not be generalizable to other caregivers, such as spouses, children-in-law, and grandchildren. Third, although the statistical methods were appropriate, no other studies have examined the FS-ADS using different methods and different populations. Fourth, the FS-ADS was not a theory-driven instrument but was designed on the basis of exploratory findings [3]. Last, the FS-ADS contains a substantial number of items, making it inconvenient for clinical use. Although using more items can capture more information, clinicians may want to use a short, quick, and cost-effective questionnaire. Therefore, we conclude that additional efforts to develop affiliate stigma instruments for caregivers of people with dementia are needed.

Instead of using the FS-ADS, we used the Affiliate Stigma Scale for caregivers of people with dementia. Although the Affiliate Stigma Scale has never been examined or used to analyze caregivers of people with dementia, it contains the following strengths. First, its psychometric properties have been examined using different methods, including classical test theory and Rasch analysis [8, 15]. Second, evidence shows that it is valid, feasible, and applicable for different populations, including people with intellectual disabilities, people with schizophrenia, and people with mood disorders [8, 15]. Thus, healthcare providers can use the Affiliate Stigma Scale to compare the affiliate stigma between caregivers of people with dementia and other mental disorders after its psychometric properties are established for caregivers of people with dementia. Third, the Affiliate Stigma Scale was designed on the basis of cognitive behavioral theory, and the mechanism of self-stigma was examined using empirical data [16].

In the present study, we used different statistical methods to examine the psychometric properties of the Affiliate Stigma Scale on a sample of caregivers who care for a family member with dementia. Specifically, internal consistency, construct validity, and concurrent validity were examined.

## Methods

This study was approved by the institutional review board of Chang Gung Memorial Hospital (IRB 102-3378B) and was done between October 2013 and September 2014. All participants were volunteers, and all participants signed informed consent forms after they felt that they understood the study purpose.

### Participants

Participants were the caregivers of outpatients at a general hospital (Chang Gung Memorial Hospital in Chiayi). Each participant had at least one family member diagnosed with dementia (including AD and vascular dementia), and the psychiatric diagnosis was based on the criteria of the *Diagnostic and Statistical Manual of Mental Disorders, Fourth Edition, Text Revision* [17]. The inclusion criteria were (1) aged 20 years or older, (2) having an elderly (≥65 years) family member diagnosed with dementia (any subtype), and (3) having clear consciousness and full understanding of Mandarin or Taiwanese Chinese. Caregivers diagnosed with a mental illness were excluded.

### Instruments

After the psychiatrists explained the study purpose to the participants, several research assistants helped those who wished to participate to complete the following questionnaires: the Affiliate Stigma Scale, the Caregiver Burden Inventory, the Taiwanese Depression Questionnaire, the Beck Anxiety Inventory, the 28-item World Health Organization Quality of Life questionnaire (WHOQOL-BREF), and one background information sheet.

#### Affiliate Stigma Scale

The Affiliate Stigma Scale was originally developed to assess the self-stigma of a caregiver providing care to a family member with a mental illness or intellectual disability [8]. Because we recruited caregivers of a family member with dementia in this study, we used the term *dementia* instead of *mental illness* or *intellectual disability* in the Affiliate Stigma Scale. This instrument has 22 items rated on a 4-point Likert scale with three domains (cognitive = 7 items, affect = 7 items, and behavior = 8 items); a higher score indicates a higher level of affiliate stigma. The psychometric properties of the Affiliate Stigma Scale have been supported, including excellent internal consistency (α = 0.85–0.94), person separation reliability (coefficient = 0.88–0.99), predictive validity, and concurrent validity [8, 15].

#### Caregiver Burden Inventory

The 24-item Caregiver Burden Inventory includes five dimensions (burdens) (time-dependent = 5 items, developmental = 5 items, physical = 4 items, social = 4 items, and emotional = 6 items). Each item was rated on a 5-point Likert scale; a higher score indicates a higher level of caregiver burden [18]. In addition, the Caregiver Burden Inventory has been translated into Taiwanese Mandarin Chinese with excellent internal consistency (α = 0.91) and appropriate content validity [19].

#### Taiwanese Depression Questionnaire

The 18-item Taiwanese Depression Questionnaire assesses the degree of depression; each item is rated from 0 (no or extremely few, <1 day per week) to 3 (often or always, 5–7 days per week). A higher score indicates a higher level of depression; the internal consistency of this instrument is satisfactory (α = 0.90) [20].

#### Beck Anxiety Inventory

The 21-item Beck Anxiety Inventory evaluates a patient’s degree of clinical anxiety; each item is rated from 0 (not at all) to 3 (severe [“I could barely stand it”]). A higher score indicates a higher level of anxiety [21], and its translated Taiwanese Mandarin Chinese version has excellent internal consistency (α = 0.95) and known-group validity [22].

#### WHOQOL-BREF

The WHOQOL-BREF Taiwan version measures quality of life (QoL). Each item is rated on a 5-point Likert scale. Except for 2 general items, the other 26 items are distributed into four domains: physical (7 items), psychological (6 items), social (4 items), and environmental (9 items). A higher score indicates a higher QoL [23]. In addition to its good psychometric properties in the general Taiwanese population (α = 0.70–0.77), the WHOQOL-BREF had satisfactory reliability and validity in populations with psychiatric problems [24, 25], with cancer [26], and with geriatric problems [27].

### Statistical analysis

The demographics and the item scores of the Affiliate Stigma Scale were analyzed using descriptive analyses such as mean, SD, and frequency. Principal component analysis (PCA) was done to determine the unidimensionality of each domain of the Affiliate Stigma Scale and for the entire scale. Rasch analysis and confirmatory factor analysis (CFA) were also used to test the misfit items and construct validity, respectively, after the unidimensionality of the Affiliate Stigma Scale was determined. When the PCA results indicate that the first component’s eigenvalue is <2, the entire Affiliate Stigma Scale fulfills the unidimensional assumption [15, 28]. Both Rasch analysis and CFA use the 22 items as a whole to examine construct validity. In contrast, if the entire Affiliate Stigma Scale is not unidimensional (i.e., if the first component’s eigenvalue is >2), both Rasch analysis and CFA examine the construct validity for each domain.

Rasch analysis with the partial credit model was used to identify misfit items. A misfit item has either an infit MnSq or an outfit MnSq that has fallen out of the 0.5–1.5 range [29, 30]. CFA with robust diagonal weighted least squares (DWLS) was used to test the construct validity of the Affiliate Stigma Scale. We used the robust DWLS estimator because of the categorical indicators in the Affiliate Stigma Scale (i.e., the 4-point Likert scale). Four fit indices in addition to the χ^2^ test were used to examine the data-model fit. The Comparative Fit Index (CFI) and Tucker-Lewis index (TLI) (>0.95), the root mean square error of approximation (RMSEA) (<0.05), and the weighted root mean square residual (WRMR) (<0.9) indicate an excellent fit [31, 32]; a CFI and TLI >0.9, an RMSEA <0.08, and a WRMR <1.0 indicate an acceptable fit [33, 34].

Moreover, internal consistency was evaluated using Cronbach’s α; a value >0.7 indicates good internal consistency. Corrected item-total correlation was computed, and a value >0.4 indicates that the item is adherent to other items measured using the same latent construct. In addition, Pearson correlations were used to analyze the concurrent validity of the Affiliate Stigma Scale. Concurrent validity is also important to understanding the validity of an instrument, and it investigates the relationship between the target instrument (i.e., the Affiliate Stigma Scale) and other existed instruments (which we call *criteria*: Caregiver Burden Inventory, Taiwanese Depression Questionnaire, Beck Anxiety Inventory, and WHOQOL-BREF in our study). The reason why we chose these instruments together as the criteria is that, on the basis of our literature review [3, 15, 35], the affiliate stigma would be positively correlated with the caregiver burden, depression, and anxiety but negatively correlated with QoL. Therefore, if we find associations between the Affiliate Stigma Scale and the criteria, we can claim satisfactory concurrent validity for the Affiliate Stigma Scale. Also, these instruments were well developed and have sound psychometric properties [18–27].

All the statistical analyses were done using the R package. Specifically, the PCAs were done using the prcomp function, the Rasch models using the eRm package [36], CFA using the lavaan package [37], Cronbach’s α and corrected total-item correlation using the CTT package [38], and Pearson correlation analysis using the Hmisc package [39].

## Results

The mean age of the caregivers was 52.80 ± 12.18 years, and their duration of caregiving was 2.45 ± 2.43 years, with an average of 10.09 ± 9.34 care hours per day (Table 1). There were slightly more female (*n* = 144) than male caregivers (*n* = 127). About one-third of the caregivers (*n* = 92) had completed junior high school or less; about one-third (*n* = 86) had completed senior high school (*n* = 86); and about one-third (*n* = 93) had completed 1 year or more of college. Three-fourths (*n* = 206) of the caregivers were married, and more than half (*n* = 167; 61.6 %) were a child of the patient. Most of the caregivers (*n* = 193; 71.2 %) were living with the patients, and most were the primary caregiver (*n* = 227; 83.8 %). Slightly more than half of the caregivers had a full-time or part-time job (*n* = 146).

**Table 1:** Demographics of participants (*n* = 271)

	Mean ± SD or *n* (%)
Age, years	52.80 ± 12.18
Duration of caregiving, years	2.45 ± 2.43
Caring time per day, h	10.09 ± 9.34
Male sex	127 (46.9 %)
Education, junior high school or less	92 (33.9 %)
Marital status, married	206 (76.0 %)
Relationship with the patient, child	167 (61.6 %)
Living with the patient, yes	193 (71.2 %)
Primary caregiver, yes	227 (83.8 %)
Otherwise employed full- or part-time, yes	146 (53.9 %)

The internal consistency was high in each domain (α = 0.822–0.855) and on the entire scale (α = 0.929). PCA showed that the first component’s eigenvalue for the entire Affiliate Stigma Scale was 2.34, but that the eigenvalues for each separate domain each were all <2 (cognitive = 0.96; affect = 1.10; and behavior = 1.18). Therefore, the following Rasch models and CFA analyses were used for separate cognitive, affective, and behavioral domains. The corrected item-total correlations and standardized factor loadings of all items were >0.4, except for the loading of item A6 (“I worry whether others know my family member has dementia”; loading = 0.394); the infit and outfit MnSq of all items were between 0.5 and 1.5, except for item C4 (“Having a family member with dementia negatively affects me”; infit MnSq = 1.57, outfit MnSq = 1.51) (Table 2).

**Table 2:** Reliability and item characteristics: number of respondents per item, corrected item-total correlation, standardized factor loadings from confirmatory factor analysis, infit and outfit MnSq from Rasch analysis, and mean scores

Domain	(Cronbach’s α)						
Item	Content	*n*	Item-total correlation	Factor loading	Infit MnSq	Outfit MnSq	Mean score
Cognitive	(0.855)						1.42
C1	Others will discriminate against me if I am with my family member with dementia	270	0.581	0.626	0.99	1.08	1.33
C2	My reputation is damaged because I have a family member with dementia at home	271	0.605	0.683	0.84	0.68	1.24
C3	People’s attitude toward me turns sour when I am with my family member with dementia	271	0.600	0.671	1.00	1.11	1.38
C4	Having a family member with dementia negatively affects me	271	0.483	0.500	1.57	1.51	1.83
C5	Having a family member with dementia makes me think that I am incompetent compared with others	271	0.767	0.796	0.61	0.61	1.45
C6	Having a family member with dementia makes me think that I am lesser than others	271	0.763	0.782	0.58	0.55	1.39
C7	Having a family member with dementia makes me lose face	271	0.705	0.785	0.63	0.51	1.29
Affective	(0.849)						1.88
A1	I feel inferior because one of my family member has dementia	271	0.448	0.445	1.08	1.10	1.47
A2	I feel emotionally disturbed because of my family member with dementia	271	0.707	0.790	0.83	0.82	2.22
A3	The behavior of my family member with dementia embarrasses me	271	0.594	0.635	0.95	0.89	1.79
A4	I feel helpless because I have a family member with dementia	270	0.657	0.726	0.81	0.93	1.90
A5	I feel sad because I have a family member with dementia	271	0.703	0.775	0.79	0.84	2.08
A6	I worry whether others know my family member has dementia	271	0.416	0.394	1.04	0.95	1.33
A7	I am under great stress because of my family member with dementia	271	0.722	0.811	0.81	0.79	2.37
Behavior	(0.822)						1.53
B1	I avoid communicating with my family member with dementia	271	0.401	0.435	1.14	1.47	1.47
B2	I dare not tell others that my family member has dementia	270	0.502	0.567	0.92	1.02	1.37
B3	I have cut down on going out with my family member with dementia	270	0.518	0.582	1.02	1.13	1.58
B4	Because of my family member with dementia, I have reduced my contacts with friends and relatives	271	0.557	0.576	0.98	0.99	1.71
B5	When I am with my family member with dementia, I keep an especially low profile	271	0.569	0.646	1.00	1.03	1.85
B6	I have reduced my contacts with my family member with dementia	271	0.648	0.725	0.63	0.63	1.37
B7	I dare not participate in activities related to dementia lest others suspect that my family member has dementia	271	0.641	0.716	0.67	0.74	1.38
B8	Because of my family member with dementia, I have reduced my contacts with my neighbors	271	0.555	0.567	0.90	0.84	1.53

All fit indices of CFA indicated satisfactory data-model fit in both the cognitive and the affective domains: a nonsignificant χ^2^ value, CFI and TLI >0.95, RMSEA <0.05, and WRMR <0.9. Although the χ^2^ value was significant in the behavioral domain, part of its fit indices were excellent (CFI and TLI >0.95) and part of them were close to acceptable (RMSEA = 0.055 and WRMR = 1.006) (Table 3).

**Table 3:** Goodness-of-fit indices of confirmatory factor analysis

	Cognitive	Affective	Behavioral
χ^2^ (*df*)	21.91 (14)	18.76 (14)	34.47 (20)^a^
Comparative Fit Index	0.991	0.996	0.974
Tucker-Lewis index	0.986	0.993	0.963
Root mean square error of approximation	0.043	0.036	0.055
Weighted root mean square residual	0.864	0.818	1.006

^a^ *p* < 0.05

The Affiliate Stigma Scale scores (including each domain score and the entire scale score) were positively and significantly correlated with the caregiver burden (*r* = 0.290–0.628), depression (*r* = 0.391–0.612), and anxiety (*r* = 0.367–0.467) and negatively and significantly correlated with QoL (*r* = −0.590 to −0.365) (Table 4).

**Table 4:** Concurrent validity of Affiliate Stigma Scale

	Affiliate Stigma Scale domains			
	Cognitive	Affective	Behavioral	Entire scale
Caregiver burden				
Time	0.290	0.488	0.351	0.429
Developmental	0.466	0.589	0.493	0.577
Physical	0.366	0.546	0.358	0.481
Social	0.373	0.416	0.434	0.452
Emotional	0.566	0.577	0.565	0.628
Depression	0.407	0.612	0.391	0.536
Anxiety	0.435	0.462	0.367	0.467
Quality of life				
Physical	−0.436	−0.491	−0.365	−0.482
Psychological	−0.457	−0.590	−0.479	−0.570
Social	−0.448	−0.464	−0.424	−0.494
Environment	−0.420	−0.445	−0.390	−0.464

All *p* values <0.001

## Discussion

Because little is known about stigma in caregivers of people with dementia, we wanted to validate an instrument that is useful and feasible for healthcare providers when assessing affiliate stigma. We also wanted that instrument to be useful for assessing the affiliate stigma of caregivers of a family member across different diseases (e.g., schizophrenia, dementia, and intellectual disability). We therefore examined the psychometric properties of the Affiliate Stigma Scale with caregivers of people with dementia, and our findings were promising. The internal consistency, construct validity, and concurrent validity of the Affiliate Stigma Scale were all satisfactory. However, on the basis of Rasch analysis, one item in the cognition domain was slightly misfit to the latent construct of which it was a member.

Our psychometric results showing that the scale was internally consistent as a whole and concurrently valid are in agreement with the findings of Mak and Cheung [8] and Chang et al. [15]. The slight difference between our results and those of Mak and Cheung [8] is in the dimensionality. Mak and Cheung suggested a one-factor solution for the entire Affiliate Stigma Scale based on a Cattell scree plot, while our PCA results indicated that there is more than one factor in the Affiliate Stigma Scale. We also found that each domain was unidimensional, which is consistent with the findings of Chang et al. [15]. In addition to the statistical views of Chang et al. [15] to support the unidimensionality in the domain but not the entire scale level, we hypothesized a theory-driven perspective that affiliate stigma follows the cognitive behavioral model [6, 16]. Moreover, although the FS-ADS was developed using exploratory instead of theory-driven methods, Werner et al. [3] also reported that their results corresponded to cognitive behavioral theory. That is, affiliate stigma is very likely to have three correlated parts. Thus, it is reasonable to find that the three domains were separate but mutually correlated in our psychometric results.

Similarly to the Rasch results of Chang et al. [15], we also found that, except for item C4 (“Having a family member with dementia imposes a negative impact on me”), all items fit well in the domain of which they were a member. One possible explanation for the misfit is that caregivers express some negative emotions when they answer “negative impact” questions. Therefore, item C4 might connote some affective meanings for the respondents. However, because the evidence is not strong, researchers in future studies might want to probe more deeply into this.

Our concurrent validity results showed that affiliate stigma was correlated with depression, caregiver burden, and QoL. It is suggested that caregivers are responsible for managing the signs and symptoms of their family member with dementia [40]; therefore, their emotional distress and caregiver burden are likely to be high, and, consequently, they are likely to have a low QoL [41, 42]. At the same time, the caregivers themselves might endorse the public stigma [35], which would result in a high level of affiliate stigma. Thus, we hypothesize a positive relationship between affiliate stigma, depression, and caregiver burden and a negative relationship between affiliate stigma and QoL. Another possibility is that the high depression and low QoL are caused by high affiliate stigma, as reported in other studies [43, 44]. However, the mechanism for these outcome factors requires additional exploration.

Our study has some limitations. First, we did not use the FS-ADS as the criterion for testing concurrent validity. Although we used several different criteria, we felt that the best standard for concurrent validity is the FS-ADS. However, because currently there is no Chinese version of the FS-ADS, we were unable to use it. In addition, the psychometric properties of the FS-ADS have been examined only for patients with AD. Given the high prevalence of vascular dementia in Taiwan [45, 46], we cannot be certain that the FS-ADS is suitable for all patients with dementia in Taiwan. Nevertheless, researchers in future studies might want to translate the FS-ADS into Chinese and cross-validate the FS-ADS and the Affiliate Stigma Scale in patients with different types of dementia. Second, because all participants were recruited from the same area in Taiwan, they might not be representative of Taiwan’s general population of patients with dementia. Because our results are comparable to those of other studies [8, 15], we feel that our results are not seriously biased. Third, we did not examine the test-retest reliability of the Affiliate Stigma Scale. Thus, the stability of the Affiliate Stigma Scale over time is unknown for caregivers of people with dementia. Fourth, although our sample size seemed sufficient for our analyses (Rasch analysis needs 250 respondents to produce reasonable estimate [47]; CFA needs at least of 200 respondents [25, 48]), we considered that our sample size was relatively small (*n* = 271) because it was only slightly over the minimum requirement. Future studies using a large sample size (say, over 500) are warranted to corroborate our psychometric findings. Fifth, our relatively small sample size consisted of various types of dementia. Specifically, dementia includes acute or chronic, degenerative or nondegenerative, reversible or irreversible, gene-causative or noncausative, and different types or etiologies may introduce different levels of stigma for the caregiver. Therefore, we should consider the influence of heterogeneity in dementia. For example, familial AD, as compared with other types of dementia, may make family members have a high level of affiliate stigma in traditional Chinese culture. However, we justified that based on our clinical experience, most family members experience stigma that is due mainly to the dementia diagnosis and the associated symptoms, regardless of the type of dementia. Therefore, we believe that our heterogeneous sample may not have seriously biased our findings.

## Conclusions

The Affiliate Stigma Scale is a reliable and valid instrument for assessing affiliate stigma. Because the psychometric properties of the Affiliate Stigma Scale have been found satisfactory, healthcare providers might want to use it to understand caregivers’ perspectives and to design appropriate interventions to reduce affiliate stigma.

## Abbreviations

AD: Alzheimer’s disease
CFA: Confirmatory factor analysis
CFI: Comparative Fit Index
DWLS: Diagonal weighted least squares
FS-ADS: Family Stigma in Alzheimer’s Disease Scale
PCA: Principal component analysis
QoL: Quality of life
RMSEA: Root mean square error of approximation
TLI: Tucker-Lewis index
WHOQOL-BREF: 28-item World Health Organization Quality of Life questionnaire
WRMR: Weighted root mean square residual
